# Orbital Angular Momentum (OAM) of Rotating Modes Driven by Electrons in Electron Cyclotron Masers

**DOI:** 10.1038/s41598-017-03533-y

**Published:** 2017-06-13

**Authors:** Ashwini Sawant, Mun Seok Choe, Manfred Thumm, EunMi Choi

**Affiliations:** 1 Department of Electrical Engineering, Ulsan National Institute of Science and Technology (UNIST), Ulsan, 44919 South Korea; 2 Department of Physics, Ulsan National Institute of Science and Technology (UNIST), Ulsan, 44919 South Korea; 3 Institute for Pulsed Power and Microwave Technology (IHM), Karlsruhe Institute of Technology (KIT), Karlsruhe, 76131 Germany

## Abstract

The well-defined orbital angular momentum (OAM) of rotating cavity modes operating near the cutoff frequency excited by gyrating electrons in a high-power electron cyclotron maser (ECM)—a gyrotron—has been derived by photonic and electromagnetic wave approaches. A mode generator was built with a high-precision 3D printing technique to mimic the rotating gyrotron modes for precise low-power measurements and shows clear natural production of higher-order OAM modes. Cold-test measurements of higher-order OAM mode generation promise the realization towards wireless long-range communications using high-power ECMs.

## Introduction

The first evidence of light beams carrying orbital angular momentum (OAM) was discovered by Allen^1^ in 1992, when a Hermite–Gaussian beam was transformed into Laguerre–Gaussian (LG) mode. It was concluded that a light beam, having an azimuthal angular dependence of exp(−*ilϕ*), carries an angular momentum of $l\hat{h}$ per photon^2, 3^. Since then, this property of OAM light beams has been widely employed in many applications such as optical tweezers, the optical drives of micromachines, atom trapping, and optical communication^4–7^. Recently, in the field of communication, the significance of OAM beams has become of great interest owing to the availability of infinite orthogonal modes, which can carry independent information over a single transmission channel^8–10^. This provides additional dimension to multiplexing techniques based on OAM beams, which can be employed in addition to conventional multiplexing techniques to achieve higher data rates^8^. The challenge was to design a channel that can exploit the possibilities of infinite orthogonal OAM modes. Wang *et al*. reported a scheme for terabit free-space data transmission at 193.4 THz by multiplexing four OAM beams^8^.

OAM beams have been studied in the optical regime, and more recently, research has focused in the millimeter-wave region^11, 12^. In this frequency band, there are difficulties associated with the beam-splitting and beam-combining processes, as the wavelength is much larger compared to those in optical frequency range. Thus, diffraction is also a critical issue, as it limits the beams to propagate for longer distances. Recently, 60 GHz wireless transmission has been realized using two OAM modes generated by a dual OAM antenna^13^. Further, multiple methods for generating OAM beams have been reported, such as a spiral phase plate^14, 15^ and computer-generated hologram^16, 17^. A few featured antennas have also been introduced to generate OAM beams^18^.

In this work, we introduce the natural generation of high-power OAM modes excited by gyrating electrons occurring in electron cyclotron masers (ECMs), i.e., gyrotrons. For precise low-power amplitude and phase measurements, we use a low-power quasi-optical (QO) mode generator^19–21^ that can convert an incident paraxial Gaussian beam into rotating transverse electric (TE) modes acting as OAM beams in free space. The advantage of this technique is the simple and natural generation of higher-order OAM modes.

In the next sections, the theory and formalism of the OAM of rotating TE modes in high-power gyrotrons are presented using photonic and electromagnetic wave approaches. Further, the design of a QO-mode generator for the low-power generation of typical rotating gyrotron TE modes is explained along with the fabrication process. The converted TE modes are then scanned in free space, and their phase patterns are analyzed to validate the OAM mode generation.

### Excitation of orbital angular momentum in electron cyclotron masers

**Figure 1:**
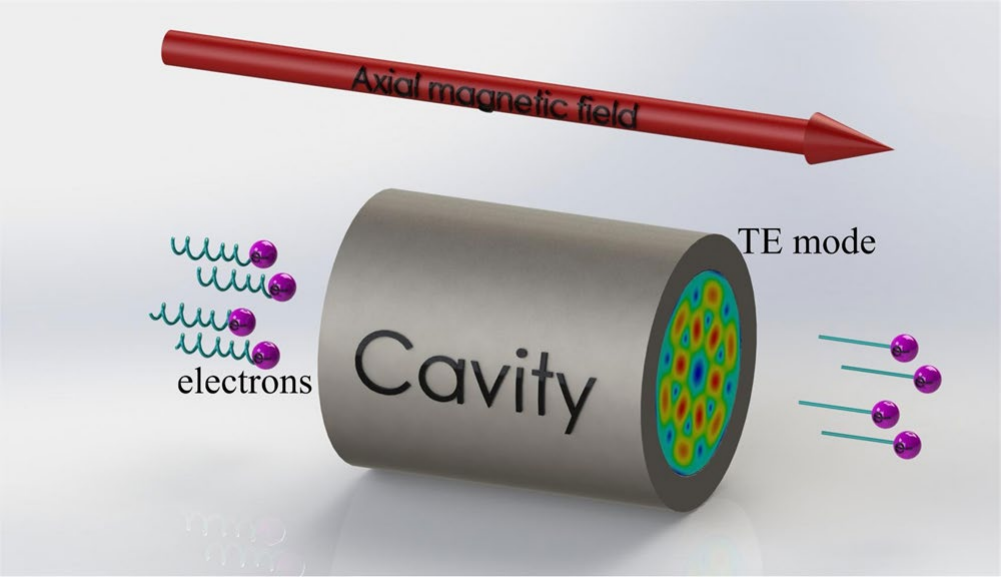
Beam–wave interaction in electron cyclotron masers.

ECMs are prominent sources of high-power RF waves in vacuum electronics. The generation of electromagnetic radiation from gyrating electrons requires bunching and synchronism^22^. During the propagation of the helically moving electrons through the interaction structure, the transverse motion of a few electrons is opposite to the direction of the RF field so that the electrons become accelerated while the remaining electrons are decelerated, which leads to electron bunching. The bunched electrons lose their energy to the RF field if they are properly synchronized. A simple illustration of electron cyclotron resonance is shown in Fig. 1. In the presence of an axial magnetic field, a DC electron beam contains gyrating electrons propagating through an interaction structure such as a cavity. In the interaction structure, the electrons interact with the RF field residing within it, and a net transfer of energy from the electrons to the RF field due to bunching takes place. The transverse energy of the electrons is transferred to the TE RF field, and the electrons exiting the interaction structure have an effective reduction in their transverse velocity while their axial velocity remains almost unaffected. In the case of gyrotrons, where the interaction structure is an open-ended cylindrical cavity, the transverse energy of the electrons is converted into a rotating cavity TE mode near the cutoff frequency, as shown in Fig. 1. Here, we will show that the generated high-power TE mode excited by electrons in an ECM possesses a well-defined OAM-mode that can be used for long-range wireless communications. The free-space communication distance is determined by the transmitter output power, frequency, and atmospheric conditions such that the gyrotron has an advantage due to its high-output-power capability. A gyrotron output power of 1 MW (90 dBm) at 100 GHz allows a communication distance up to kilometers, which is dependent on the sensitivity of the receiver. Here, the free-space transmission loss due to an absolute humidity of 0.5 *g*/*m* ^3^, which is 2.1 dB/km at 100 GHz, has been taken into account. The corresponding attenuations at 400 GHz and 1 THz are 10 dB/km and 20 dB/km, respectively^23^. Furthermore, the detectable distance can be substantially increased using a high-gain focusing lens and receiving antenna. To achieve high-frequency stability and a narrow linewidth, a phase locking system can be used to control the electron beam energy^24^. The long-term stability can be guaranteed by a reference clock. A line width as narrow as 1 Hz can be obtained. However, such application will require gyrotrons with specific features. One essential characteristic for OAM gyrotrons is the axial RF output configuration, where the cavity modes will be directly emitted into free space through the RF output window rather than converting it into a Gaussian beam, as is done for high-power gyrotrons for fusion plasma heating. The propagation of OAM beams generated by gyrotrons in free space results in a large diffraction angle, which can be compensated by a waveguide diameter taper section that is added after the gyrotron cavity to reduce the Brillouin angle of the output wave and by employing a focusing lens to reduce diffraction to a certain limit.

### Orbital angular momentum of rotating modes

#### Photonic approach

**Figure 2:**
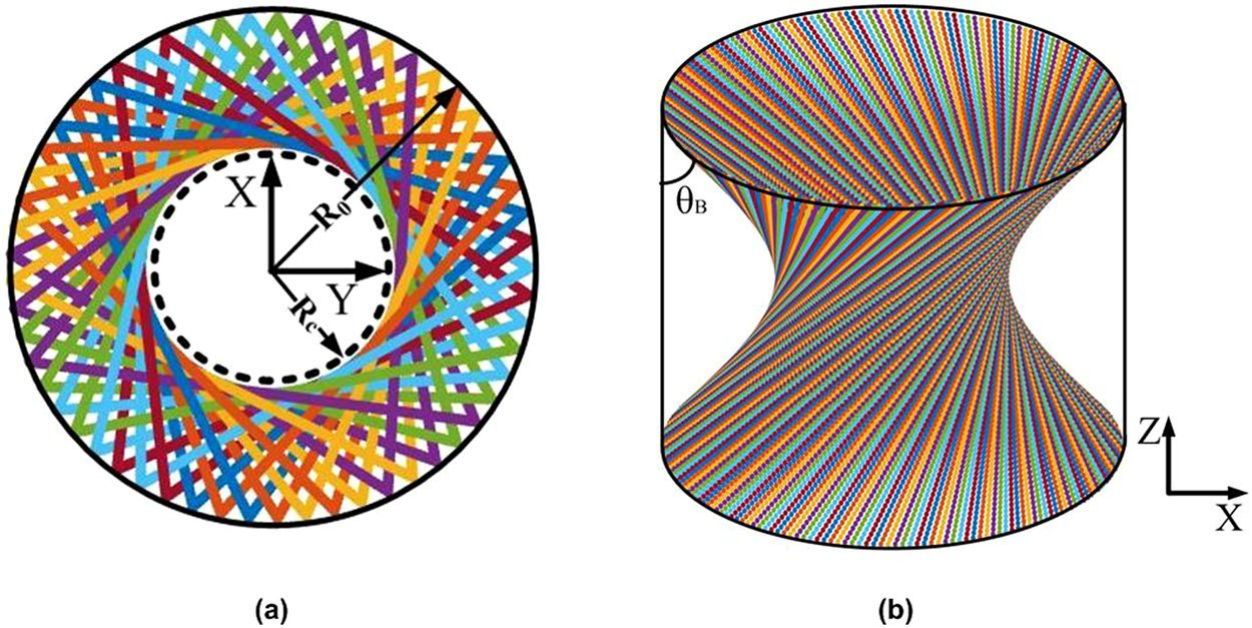
(**a**) Ray propagation for a rotating wave in a cylindrical gyrotron cavity with consecutive reflections. (**b**) Set of rays forming a caustic with radius *R*_*c*_ = $\frac{m}{{\chi }_{m,p}}$ *R*_0_.

In the geometrical optical limit, a plane wavefront is represented by one ray^25^. The helically propagating rotating TE_m,p_ modes in a circular waveguide can be decomposed into a series of cylindrical plane waves, each propagating at the Brillouin angle *θ*_*B*_ relative to the waveguide axis as shown in Fig. 2. The requirement of a zero azimuthal electric field at the waveguide wall defines their relative phases. Transverse electric field is defined by the requirement that the ray direction must coincide with the direction of the Poynting vector^26^. For a TE_m,p_ mode, the minimum distance from the waveguide center to the rays is as follows:

1 $${R}_{c}=\frac{m}{{\chi }_{m,p}}{R}_{0}\mathrm{.}$$

where *R*_*c*_ is the caustic radius, *m* is the azimuthal mode index, *χ*_*m*,*p*_ is the p^*th*^ root of the derivative of the Bessel function ${J}_{m}^{^{\prime} }(x)$, and *R*_0_ is the waveguide radius. Hence, if all plane waves are represented by geometrical optical rays, they form a caustic at the radius *R*_*c*_. In an unperturbed circular symmetric waveguide, the density of the rays along the caustic is uniform (see Fig. 2). The angular momentum $\mathop{L}\limits^{\longrightarrow}$ possessed by a ray can be expressed as

2 $$\vec{L}=L\hat{z}=\vec{r}\times \vec{p}={R}_{c}\hat{r}\times {p}_{\varphi }\hat{\varphi }={R}_{c}{p}_{\varphi }\hat{z},$$

where *p*_*ϕ*_ = *psinθ*_*B*_ = $\hslash$ *ksinθ*_*B*_, *sinθ*_*B*_ = $\frac{{k}_{\perp }}{k}$, and *k*_⊥_ = $\frac{{\chi }_{m,p}}{{R}_{0}}$, *p* is the transverse momentum and *k* is the wave vector.

Therefore, $\vec{L}={R}_{c}{p}_{\varphi }\hat{z}=m\hslash \hat{z}$. The ratio between the total angular momentum *L*_*N*_ and the total energy of photons *W*_*N*_ is:

3 $$\frac{{\vec{L}}_{N}}{{W}_{N}}=\frac{Nm\hslash }{N\hslash \omega }=\frac{m}{\omega },$$

where *ω* is the angular frequency of the resonating mode.

#### Electromagnetic wave approach

In a cylindrical coordinate system (*r*, *ϕ*, *z*), the electric and magnetic fields of the rotating TE_m,p_ modes can be expressed as follows:

4 $${E}_{r}=j{E}_{1}\frac{m}{{k}_{\perp }r}{J}_{m}({k}_{\perp }r){e}^{-jm\varphi }f(z),$$

5 $${E}_{\varphi }={E}_{1}{J}_{m}^{^{\prime} }({k}_{\perp }r){e}^{-jm\varphi }f(z),$$

6 $${E}_{z}=\mathrm{0,}$$

7 $${H}_{r}=-j{E}_{1}\frac{1}{\omega {\mu }_{0}}{J}_{m}^{^{\prime} }({k}_{\perp }r){e}^{-jm\varphi }f^{\prime} (z),$$

8 $${H}_{\varphi }=-{E}_{1}\frac{m}{\omega {\mu }_{0}{k}_{\perp }r}{J}_{m}({k}_{\perp }r){e}^{-jm\varphi }f^{\prime} (z),$$

9 $${H}_{z}=-j{E}_{1}\frac{{k}_{\perp }}{\omega {\mu }_{0}}{J}_{m}({k}_{\perp }r){e}^{-jm\varphi }f(z),$$

where *J*_*m*_(*x*) is the Bessel function of first kind and order m and ${J}_{m}^{^{\prime} }(x)$ is its derivative. The angular momentum density of the electromagnetic field can be expressed as $\vec{l}=l\hat{z}=\vec{r}\times \vec{\wp }=r{\wp }_{\phi }\hat{z}$, where $\vec{\wp }$ is the linear momentum density.

The time-averaged momentum density is given by $\langle \vec{\wp }\rangle ={\mu }_{0}{\varepsilon }_{0}\langle \vec{S}\rangle =\frac{{\mu }_{0}{\varepsilon }_{0}}{2}Re(\vec{E}\times {\vec{H}}^{\ast })$, where $\langle \vec{S}\rangle$ is the time-averaged Poynting vector. Then, it follows that

10 $$\begin{array}{rcl}\langle \vec{\wp }\rangle  & = & {\mu }_{0}{\varepsilon }_{0}\langle \vec{S}\rangle =\frac{{\mu }_{0}{\varepsilon }_{0}}{2}Re(\vec{E}\times {\vec{H}}^{\ast })\\  & = & \frac{{\mu }_{0}{\varepsilon }_{0}}{2}[({E}_{r}\hat{r}+{E}_{\varphi }\hat{\varphi })\times ({H}_{r}^{\ast }\hat{r}+{H}_{\varphi }^{\ast }\hat{\varphi }+{H}_{z}^{\ast }\hat{z})]\\  & = & \frac{{\mu }_{0}{\varepsilon }_{0}}{2}[({E}_{\varphi }{H}_{z}^{\ast })\hat{r}-({E}_{r}{H}_{z}^{\ast })\hat{\varphi }+({E}_{r}{H}_{\varphi }^{\ast }-{E}_{\varphi }{H}_{r}^{\ast })\hat{z}]\mathrm{.}\end{array}$$

Therefore, the angular momentum density can be calculated as

11 $$\overrightarrow{l}=r\hat{{{\rm{\wp }}}_{\varphi }}(z)=-r\frac{{\mu }_{0}{\varepsilon }_{0}}{2}{E}_{r}{H}_{z}^{\ast }\hat{z}.$$

Since the term ${E}_{r}{H}_{z}^{\ast }$ can be expanded as

12 $$\begin{array}{rcl}{E}_{r}{H}_{z}^{\ast } & = & (j{E}_{1}\frac{m}{{k}_{\perp }r}{J}_{m}({k}_{\perp }r){e}^{-jm\varphi }f(z))\times (j{E}_{1}^{\ast }\frac{{k}_{\perp }}{\omega {\mu }_{0}}{J}_{m}({k}_{\perp }r){e}^{+jm\varphi }{f}^{\ast }(z))\\  & = & -{|{E}_{1}|}^{2}\frac{m}{\omega {\mu }_{0}r}{J}_{m}^{2}({k}_{\perp }r){|f(z)|}^{2},\end{array}$$

the total angular momentum within a given waveguide volume can be calculated by

13 $$\begin{array}{rcl}L & = & \int ldv=\int r\frac{{\mu }_{0}{\varepsilon }_{0}}{2}\times {|{E}_{1}|}^{2}\frac{m}{\omega {\mu }_{0}r}{J}_{m}^{2}({k}_{\perp }r){|f(z)|}^{2}rdrd\varphi dz\\  & = & \pi {R}_{0}^{2}{\varepsilon }_{0}\frac{1}{2}{|{E}_{1}|}^{2}\frac{m}{\omega }(1-{(\frac{m}{{\chi }_{mp}})}^{2}){J}_{m}^{2}({\chi }_{mp})\int |f(z{)|}^{2}dz\mathrm{.}\end{array}$$

Here, we used the property of Bessel functions that ${\int }_{0}^{x}x{J}_{n}^{2}(\lambda x)dx=\frac{{x}^{2}}{2}[{({J}_{n}^{^{\prime} }(\lambda x))}^{2}+(1-\frac{{n}^{2}}{{(\lambda x)}^{2}}){({J}_{n}(\lambda x))}^{2}]$, where in our case, ${J}_{m}^{^{\prime} }({k}_{\perp }{R}_{0})={J}_{m}^{^{\prime} }({\chi }_{mp})=0$.

On the other hand, the time-averaged energy stored within the same waveguide volume can be expressed as

14 $$U=\frac{{\varepsilon }_{0}}{2}\int {|E|}^{2}dv={|{E}_{1}|}^{2}{\varepsilon }_{0}\frac{\pi }{2{k}_{\perp }^{2}}({\chi }_{mp}^{2}-{m}^{2}){J}_{m}^{2}({\chi }_{mp})\int |f(z{)|}^{2}dz\mathrm{.}$$

Therefore, the total angular momentum of a ECM cavity mode within a given waveguide volume per total energy of the electromagnetic wave in the same volume can be expressed as follows:

15 $$\frac{|\overrightarrow{L}|}{U}=\frac{\pi {R}_{0}^{2}{\varepsilon }_{0}\frac{1}{2}{|{E}_{1}|}^{2}\frac{m}{\omega }(1-{(\frac{m}{{\chi }_{mp}})}^{2}){J}_{m}^{2}({\chi }_{mp})\int |f(z{)|}^{2}dz}{{|{E}_{1}|}^{2}{\varepsilon }_{0}\frac{\pi }{2{k}_{\perp }^{2}}({\chi }_{mp}^{2}-{m}^{2}){J}_{m}^{2}({\chi }_{mp})\int |f(z{)|}^{2}dz}=\frac{m}{\omega }\mathrm{.}$$

### Spin angular momentum and orbital angular momentum of gyrotron modes

**Figure 3:**
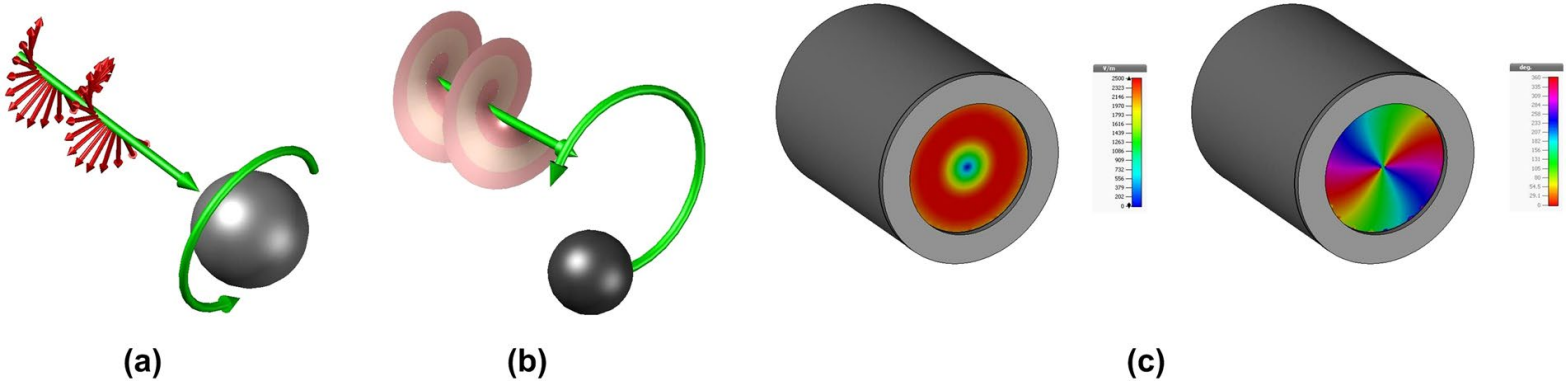
(**a**) Spin angular momentum (SAM). (**b**) Orbital angular momentum (OAM). (**c**) Amplitude and (**d**) phase of the TE_2,1_ mode.

The identical expression in Eqs 3 and 15 obtained from the photonic and electromagnetic wave approaches, respectively, for the angular momentum volume density are also valid for the TM_m,p_ modes. This reveals that the TE_0,p_ and TM_0,p_ modes have no angular momentum, whereas the TE_1,p_ and TM_1,p_ modes show some degree of linear polarization, which means that such waves exhibit a spin angular momentum (SAM) if their right-hand and left-hand rotation components are different^27^. In corrugated waveguides, they are converted into linearly polarized LP_0,n_ modes (balanced hybrid HE_1,n_ modes)^28, 29^. The higher-order TE_m,p_ and TM_m,p_ modes (*m* ≥ 2) have no intensity along the waveguide axis and therefore only exhibit an OAM. (see Fig. 3).

Since gyrotron cavities excited by electrons oscillate at frequencies that are very close to the cutoff frequency of the operating high-order cavity mode in the cylindrical cavity section, the angular frequency *ω* is $\frac{c{\chi }_{m,p}}{{R}_{0}}$, where *c* is the velocity of light. Therefore, one can redefine the total angular momentum of a gyrotron cavity mode in a given waveguide volume per total energy of the electromagnetic wave within the same volume as

16 $$\frac{|\mathop{L}\limits^{\longrightarrow}|}{U}=\frac{m}{\omega }=\frac{{R}_{c}}{c}\mathrm{.}$$

Since *c* is a constant, Eq. 16 clearly indicates that a higher OAM will be obtained for an ECM cavity mode with a larger caustic radius. OAM is directly proportional to *m* and is inversely proportional to *ω*. Therefore, higher order rotating cavity modes will enhance the OAM per energy, and frequencies lower than those of the optical regime may be beneficial.

### Generation of gyrotron OAM modes

**Figure 4:**
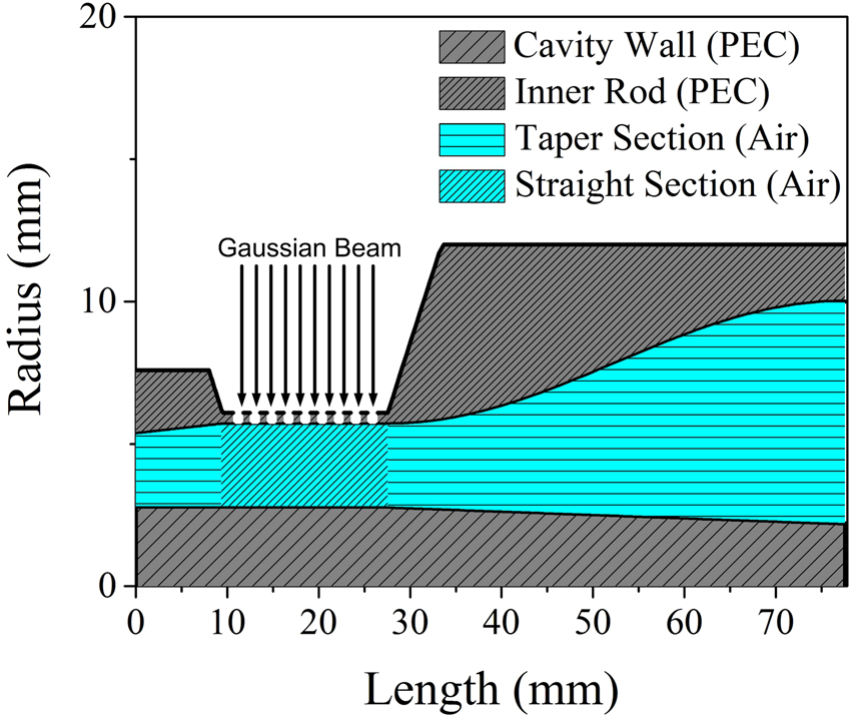
Geometry of the QO-mode generator.

**Figure 5:**
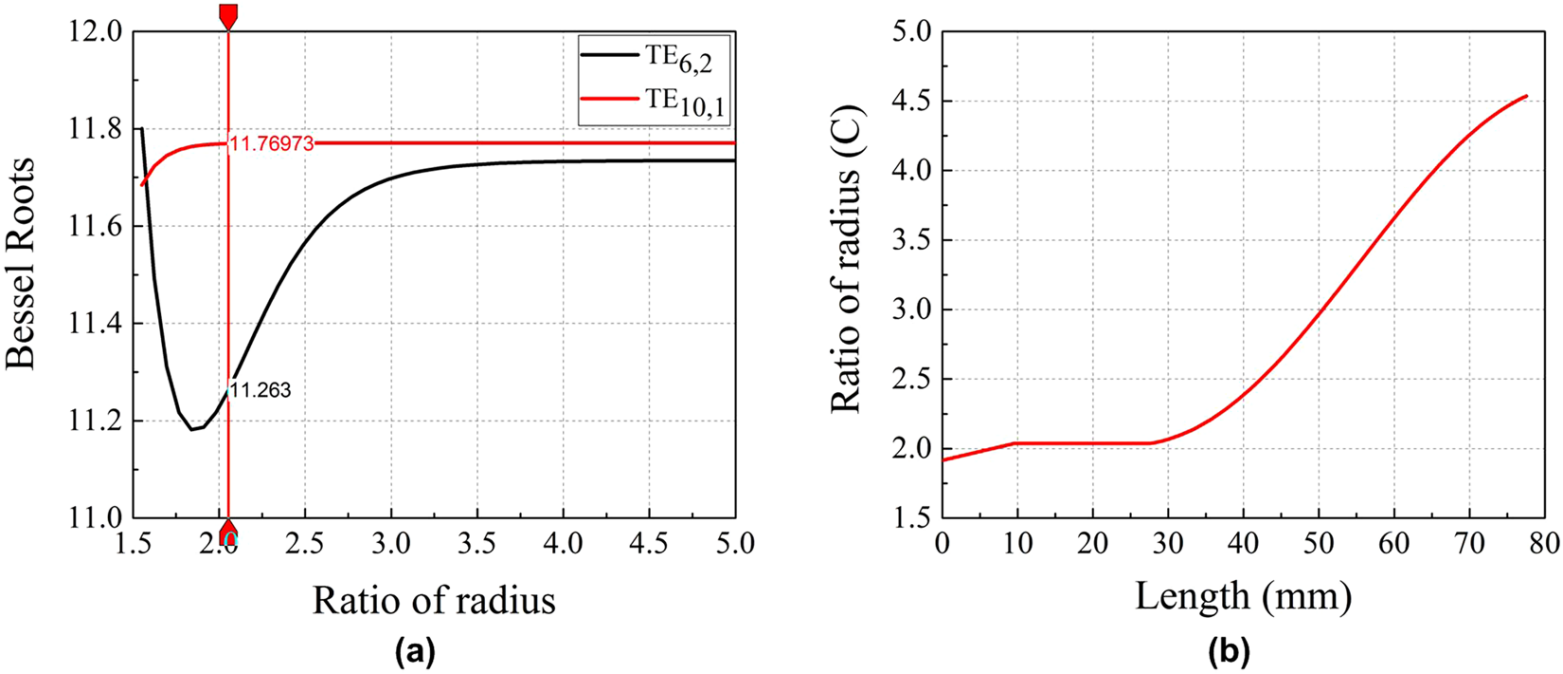
(**a**) Effective Bessel roots versus the ratio *C* of the cavity radius to the inner rod radius for the mode pair TE_6,2_ and TE_10,1_. The vertical line indicates the radii ratio at the center of the cavity. (**b**) *C* along the length of the cavity.

For precise measurements of the amplitude and phase, we generate gyrotron cavity modes by using the low-power QO-mode generator shown in Fig. 4 instead of high-power gyrotron. The capability of the rotating mode generator to mimic the cavity modes allows us to experimentally validate the dependency of the OAM on the azimuthal mode number. We have designed a dual-mode QO-mode generator capable of generating both of the TE_6,2_ and TE_10,1_ modes at the W-band frequency (≈95 GHz). It is admissible to choose a higher-order whispering gallery mode pair for our experiment, as the OAM is proportional to the azimuthal mode number [Eqs 3 and 16]. The TE_6,2_ and TE_10,1_ modes are two such competing waveguide modes whose frequencies are similar, allowing us to use a single gyrotron-like cavity and coaxial inner rod. However, their caustic radii are quite different. A coaxial waveguide structure is adopted to generate a feasible frequency difference between these two competing modes and to suppress the undesired spurious modes. The dependence of the effective Bessel roots of the coaxial cavity on the ratio *C* of the cavity radius *R*_0_ to the inner rod radius *b* for these two modes is shown in Fig. 5(a), which is calculated by following characteristic equation of a coaxial cavity^30^:

17 $${J}_{m}^{^{\prime} }(\frac{{\chi }_{m,n}}{C}){Y}_{m}^{^{\prime} }({\chi }_{m,n})-{Y}_{m}^{^{\prime} }(\frac{{\chi }_{m,n}}{C}){J}_{m}^{^{\prime} }({\chi }_{m,n})=\mathrm{0,}$$

where ${Y}_{m}^{\text{'}}(x)$ is the derivative of Bessel function of second kind and order *m*. The difference between the effective Bessel roots for the mode pair is maximized at the value of *C*  =  1.84. This finite difference in the effective Bessel roots allows for the excitation of the TE_6,2_ and TE_10,1_ modes at 94.6 and 98.6 GHz, respectively, for an optimum value of *C* = 2.037, which is indicated by the vertical line in Fig. 5(a). The variation in *C* along the cavity length is plotted in Fig. 5(b).

The diffractive quality factor *Q*_*d*_ of the resonator is calculated by UGDT (UNIST Gyrotron Design Tool, in-house developed code)^31^, which solves the Vlasov equation^32^. The calculated values of *Q*_*d*_ are 356 and 295 for the TE_6,2_ and TE_10,1_ modes, respectively. The ohmic quality factor of the resonator is defined as^19^

18 $${Q}_{ohm}=\frac{{R}_{0}}{d}\frac{(1-{(m/{\chi }_{m,n})}^{2})}{1+\frac{b}{{R}_{0}}\frac{{Z}_{m}^{2}({k}_{\perp }b)}{{Z}_{m}^{2}({k}_{\perp }{R}_{0})}},$$

where ${Z}_{m}({\chi }_{m,n}\frac{r}{{R}_{0}})={J}_{m}({\chi }_{m,n}\frac{r}{{R}_{0}}){Y}_{m}^{^{\prime} }(\frac{{\chi }_{m,n}}{C})-{Y}_{m}({\chi }_{m,n}\frac{r}{{R}_{0}}){J}_{m}^{^{\prime} }(\frac{{\chi }_{m,n}}{C})$, and *d* is the skin depth. The value of the ohmic quality factor for the optimized cavity design is *Q*_*ohm*_ = 2.26 × 10^3^ for the TE_6,2_ mode and *Q*_*ohm*_ = 1.16 × 10^3^ for the TE_10,1_ mode. To provide coupling to the resonator, the straight section of the cavity is perforated. These perforations provide power input coupling, which is described by the grid quality factor *Q*_*grid*_ dependent upon the hole dimensions:

19 $${Q}_{grid}=\frac{2\pi }{{\rm{\Theta }}}\frac{{\chi }_{m,n}(1-{(m/{\chi }_{m,n})}^{2})}{{T}^{2}},$$

where 2Θ is the azimuthal extent of the perforations in radians, and *T* ^2^ is the transmission coefficient of the wall. *T* ^2^ is calculated as reported in ref. 19, and its value is 4.51 × 10^−3^ for 0.2-mm-thick wall and 1-mm-diameter holes. The azimuthal extent of the perforations is chosen to be 360° to reduce the excitation of the counter-rotating modes. The calculated values for a 0.2-mm-thick perforated resonator wall is 1787 and 726 for the TE_6,2_ and TE_10,1_ modes, respectively. The condition for the maximum output power is as follows:

20 $$\frac{1}{{Q}_{grid}}=\frac{1}{{Q}_{d}}+\frac{1}{{Q}_{ohm}}\mathrm{.}$$

To achieve maximum power, *Q*_*grid*_ must be decreased further, which—according to Eq. 20— could be achieved by increasing the hole diameter or by decreasing the wall thickness. However, increasing the hole diameter could adversely influence the resonating properties of the cavity, whereas decreasing the wall thickness is technologically limited. Considering the machining process for fabrication, a wall thickness of 0.3 mm is a reasonable value^33^.

**Figure 6:**
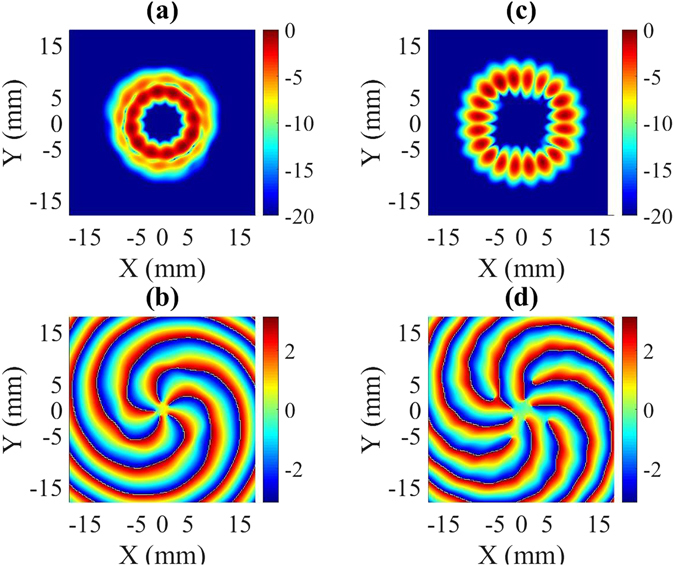
(**a**) Calculated amplitude and (**b**) phase of the time-averaged field of the TE_6,2_ mode at 94.8 GHz. (**c**) Calculated amplitude and (**d**) phase of the time-averaged field of the TE_10,1_ mode at 98.6 GHz.

The design of the mode generator is optimized using the CST Microwave Studio 2016 tool^34^. The simulation results consisting of the amplitude and phase patterns of the output electric field in the form of the TE_6,2_ and TE_10,1_ modes are shown in Fig. 6. The calculated time-averaged amplitude of the electric field in Fig. 6(a) consists of two circular rings that indicate a TE_m,2_ mode. However, *m* of the mode is difficult to identify with the amplitude pattern since the data show the time-averaged amplitude of two rings. However, *m* can be easily identified using the phase pattern of either the horizontal or vertical polarization component. The calculated phase pattern of the electric field in Fig. 6(c) contains five spiral rings, which confirms the TE_6,2_ mode [see Supplementary Information]. Moreover, at 98.6 GHz, the mode can be easily identified as the TE_10,1_ mode from the time-averaged amplitude of the electric field plotted in Fig. 6(b), as the presence of a higher content of the counter-rotating mode creates a standing pattern and 10 azimuthal variations in the amplitude can be easily observed in the amplitude pattern. The corresponding phase pattern in Fig. 6(d) also predicts the TE_10,1_ mode, as it contains nine spiral rings.

**Figure 7:**
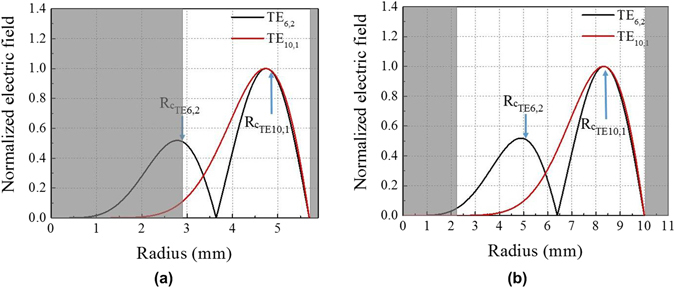
Calculated normalized electric field intensity versus the radius at (**a**) the center and (**b**) output end of the resonator. The shaded region indicates the metallic region comprising the cavity and inner rod.

The mode purity of the generated modes can be calculated from the time-averaged amplitude pattern, as reported by Wagner *et al*.^35^. The calculation predicts that the generated TE_6,2_ mode at 94.8 GHz contains 99.45% of the co-rotating mode, and the TE_10,1_ mode at 98.6 GHz contains 93.28% of the co-rotating mode. The TE_10,1_ mode contains a higher content of the counter-rotating mode because its caustic radius of 4.83 mm is much larger than the inner rod radius (*b* = 2.793 *mm*) as compared to the caustic radius of 2.91 mm of the TE_6,2_ mode, as shown in Fig. 7(a). The shaded region in Fig. 7 indicates the metallic section of the inner rod and outer wall. The inner rod radius is much closer to the caustic radius of the TE_6,2_ mode, resulting in a better mode purity compared that of TE_10,1_ mode.

### Experiment

**Figure 8:**
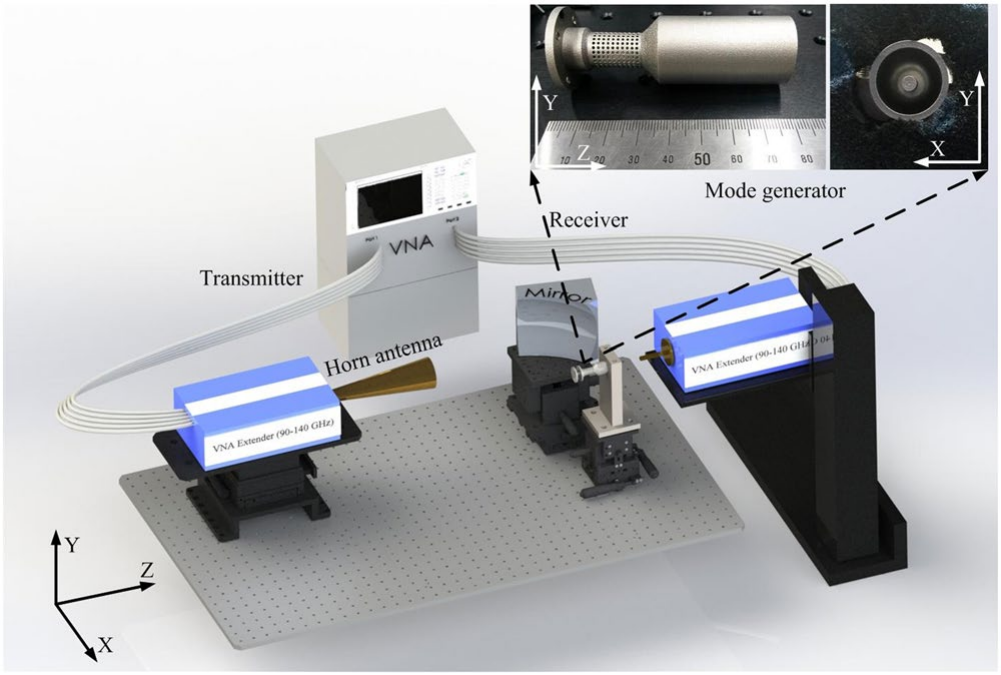
Experimental setup for the generation of rotating higher–order TE modes. Inset: QO-mode generator and inner rod.

The designed dual-mode QO-mode generator was fabricated using a commercial 3D printing technique with a precision of a few tenths of micrometers, as shown in Fig. 8. The metal used for fabrication is stainless steel SUS-3080, and in this case the minimum achievable wall thickness is 0.4 mm. This compromise has to be made owing to the application of the 3D printing technique. The surface roughness is also a critical issue in this process, and additional polishing of the internal surface was required to smoothen the wall. By polishing, the rms surface roughness of the inner wall of the mode generator was reduced to 1.25 *μm*. Precision drilling was then performed to generate holes with diameters of 1.0 mm. The inner rod was also fabricated with the 3D printing technique using stainless steel SUS-3080.

**Figure 9:**
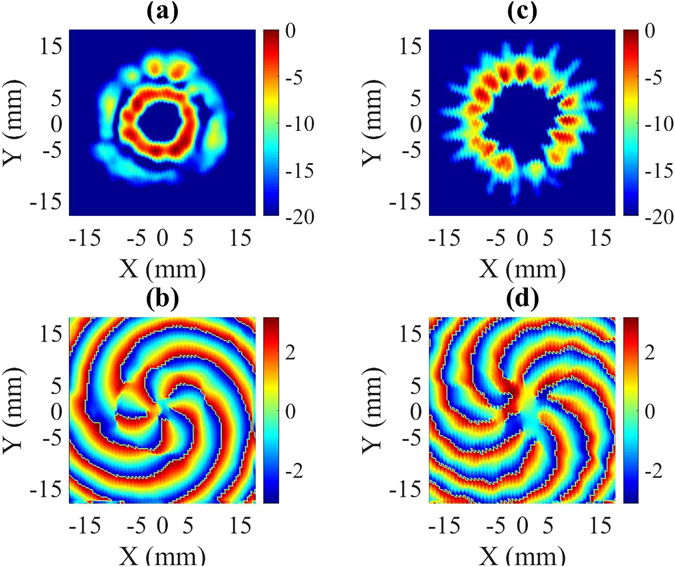
Time-averaged measured (**a**) amplitude and (**b**) phase of the electric field of the TE_6,2_ mode at 95.29 GHz and the time-averaged measured (**c**) amplitude and (**d**) phase of the electric field of the TE_10,1_ mode at 98.58 GHz.

The experimental setup for rotating higher-order TE-mode generation using the QO-mode generator is shown in Fig. 8. A vertically polarized Gaussian beam is generated by a W-band waveguide feed Gaussian horn antenna connected to a vector network analyzer (VNA; Agilent, model no. N5247A). The frequency of the output Gaussian beam is varied by changing the frequency of the incoming RF signal from the VNA. The Gaussian beam escaping the horn antenna diverges as it propagates in free space. To focus the Gaussian beam at the caustic radius of the QO-mode generator, an off-axis ellipsoidal mirror has been designed and manufactured. The mirror focuses the Gaussian beam at a distance of 10.2 mm from the mirror and changes the direction of propagation by 90°. The QO-mode generator along with the inner rod is placed such that the reflected Gaussian beam is focused 2.94 mm vertically off from its axis, which is near to the caustic of the TE_6,2_ mode. The Gaussian beam reflected from the ellipsoidal mirror passes through the translucent surface of the perforated cavity and excites the TE mode inside the QO-mode generator. The excited mode propagates along the length of the open-ended cavity and is radiated into free space from the up-taper side. The field pattern of the generated TE mode is measured by a diode detector placed on a two-dimensional translation stage and connected to the receiving port of the VNA. The electric field of the propagating field in a two-dimensional plane can be measured at different input frequency signals. The measured electric field patterns at a distance of 10 mm from the output end of the QO-mode generator at 95.29 and 98.58 GHz are shown in Fig. 9. The generated modes are whispering gallery modes, even though a small misalignment or tilt can adversely affect the mode pattern, because the inner rod radius in the perforated cavity section is very close to the caustic of the TE_6,2_ mode. This experimental setup is quite sensitive to misalignment, which can cause asymmetry in the generated mode pattern. The alignments of the inner rod and mirror are the major factors that affect the field pattern of the generated mode. The alignment of the mirror is very crucial as the reflected Gaussian beam should be focused at the caustic.

The frequency of the incoming signal was varied with a resolution of 0.01 GHz and the field pattern is scanned at a distance of 10 mm from the converter, as already mentioned. A rigorous analysis of the measured field shows that the excitation of high-purity TE_6,2_ and TE_10,1_ modes occurs at 95.29 and 98.58 GHz, respectively. The measured field patterns (time-averaged amplitude and phase) in Fig. 9 are consistent with the simulation results in Fig. 6. The amplitude and phase patterns consist of two circular rings and 5 spirals, respectively, which assures the existence of a rotating TE_6,2_ mode at 95.29 GHz. Similarly, at 98.6 GHz, the measured amplitude pattern contains a single circular ring close to the cavity wall, and the measured phase pattern contains nine spirals, which confirm the TE_10,1_ mode.

## Conclusion

We have theoretically confirmed the quantification of the OAM of high-order TE_m,n_ modes excited by gyrating electrons in the cavity of an ECM (gyrotron) by photonic and electromagnetic wave approaches. From our analysis, the momentum per energy within a given volume depends on *m* and *ω* and is simply given by $\frac{m}{\omega }$. Since the operating TE mode in a gyrotron cavity is very close to cutoff, this ratio geometrically depends only on the caustic radius of the mode, which confirms that whispering gallery modes possess a strong OAM. Experimentally, we excited two selected higher-order OAM modes (TE_6,2_ and TE_10,1_) with the aid of a low-power QO-mode generator instead of gyrating electrons at a high power in order to demonstrate the properties of the OAM modes. The measured phase patterns of the modes in free space contain an (*m* − 1) feature of the spirals, characterizing its rotating behavior. The generation of such higher-order OAM modes in the millimeter-wave regime without any insertion devices, as in optics, is unique. This conceptual interpretation of gyrotron cavity modes as OAM modes will open new applications for high-power millimeter-wave sources such as gyrotrons, where the high-power requirement at millimeter-wave frequencies is critical. Research in the field of long-range wireless communication could be an area where gyrotron OAM modes can realize greater significance.

## Electronic supplementary material

supplementary
