# Supplementary material for: Orbital Angular Momentum (OAM) of Rotating Modes Driven by Electrons in Electron Cyclotron Masers
Source: Sci Rep. 2017 Jun 13;7:3372. doi: 10.1038/s41598-017-03533-y (PMC5469808; doi:10.1038/s41598-017-03533-y)
Supplement: Supplementary file 1 — supplementary [file 41598_2017_3533_MOESM1_ESM.pdf]

Orbital Angular Momentum (OAM) of Rotating Modes Driven by Electrons in Electron Cyclotron Masers

Ashwini Sawant[1], Mun Seok Choe[2], Manfred Thumm[3], and EunMi Choi[1,2,*]

[1]Department of Electrical Engineering, Ulsan National Institute of Science and Technology (UNIST), South Korea, 44919
[2]Department of Physics, Ulsan National Institute of Science and Technology (UNIST), South Korea, 44919
[3]Institute for Pulsed Power and Microwave Technology (IHM), Karlsruhe Institute of Technology (KIT), Germany
[*]emchoi@unist.ac.kr

Supplementary Information

Phase patterns of higher-order modes

The near field of higher-order rotating $TE_{m,n}$ modes after propagation in free space contains m spirals, but its horizontal and vertical component contain $(m-1)$ spirals. The E_ϕ component of the rotating TE mode is expressed in Eq. 5 (Main article). The horizontal component E_x and vertical component E_y are as follows:

$$\begin{aligned}
E_x &= E_r cos(\phi) - E_\phi sin(\phi) \\
&= jE_1 \frac{m}{k_\perp r} J_m(k_\perp r) e^{-jm\phi} f(z) cos(\phi) - E_1 J_m^{'}(k_\perp r) e^{-jm\phi} \\
&\quad f(z) sin(\phi) \\
&= E1 f(z) \Bigg(j \frac{m}{k_\perp r} J_m(k_\perp r)(cos(m\phi) - jsin(m\phi)) cos(\phi) \\
&\quad - J_m^{'}(k_\perp r)(cos(m\phi) - jsin(m\phi)) sin(\phi) \Bigg),
\end{aligned}$$

For higher-order modes $\frac{m}{k_\perp r} J_m(k_\perp r) = J_m^{'}(k_\perp r)$ when $r \to 0$. Thus,

$$E_x = jE_1 f(z) J_m^{'}(k_\perp r) e^{-j(m-1)\phi}. \tag{1}$$

Similarly,

$$E_y = E_1 f(z) J_m^{'}(k_\perp r) e^{-j(m-1)\phi}. \tag{2}$$

Total electric field (E) can be expressed as

$$E = E_x + jE_y = j2E_1 f(z) J_m^{'}(k_\perp r) e^{-j(m-1)\phi}. \tag{3}$$

Equations 3 suggests that the electric field of the rotating higher-order mode $TE_{m,n}$ contains $m-1$ phase singularities at the center of the beam. This property of the rotating mode plays a significant role in identifying the azimuthal index of the generated mode.
